# Supplementary material for: Adenosine Triphosphate Accumulated Following Cerebral Ischemia Induces Neutrophil Extracellular Trap Formation
Source: Int J Mol Sci. 2020 Oct 16;21(20):7668. doi: 10.3390/ijms21207668 (PMC7589755; doi:10.3390/ijms21207668)
Supplement: Supplementary file 1 [file ijms-21-07668-s001.pdf]

Supplementary information

ATP induces neutrophil extracellular trap formation in the post-ischemic brain

Seung-Woo Kim[a,b,d], Dashdulam Davaanyam[a,b], Song-I Seol[a,b,c], Hye-Kyung Lee[a,b,] Hahnbie Lee[a,b], Ja-Kyeong Lee[a,b,c*]

[a]Department of Anatomy, Inha University School of Medicine, Incheon, 22212, Korea

[b]Medical Research Center, Inha University School of Medicine, Incheon, 22212, Korea

[c]Program in Biomedical Science & Engineering, Inha University School of Medicine, Incheon, 22212, Korea

[d]Department of Biomedical Sciences, Inha University School of Medicine, Incheon, 22212, Korea

Key words: Neutrophil, NETosis, stroke, ATP, PAD4, ROS

Running title: NETosis induction by ATP in the ischemic brain

***Corresponding author:**

Ja-Kyeong Lee, Ph.D.

Department of Anatomy, Medical Research Center, Inha University School of Medicine.

Inharo 100, Inchon, 22202, Republic of Korea, Tel, +82-32-860-9893; jklee@inha.ac.kr

Supplementary Figure 1

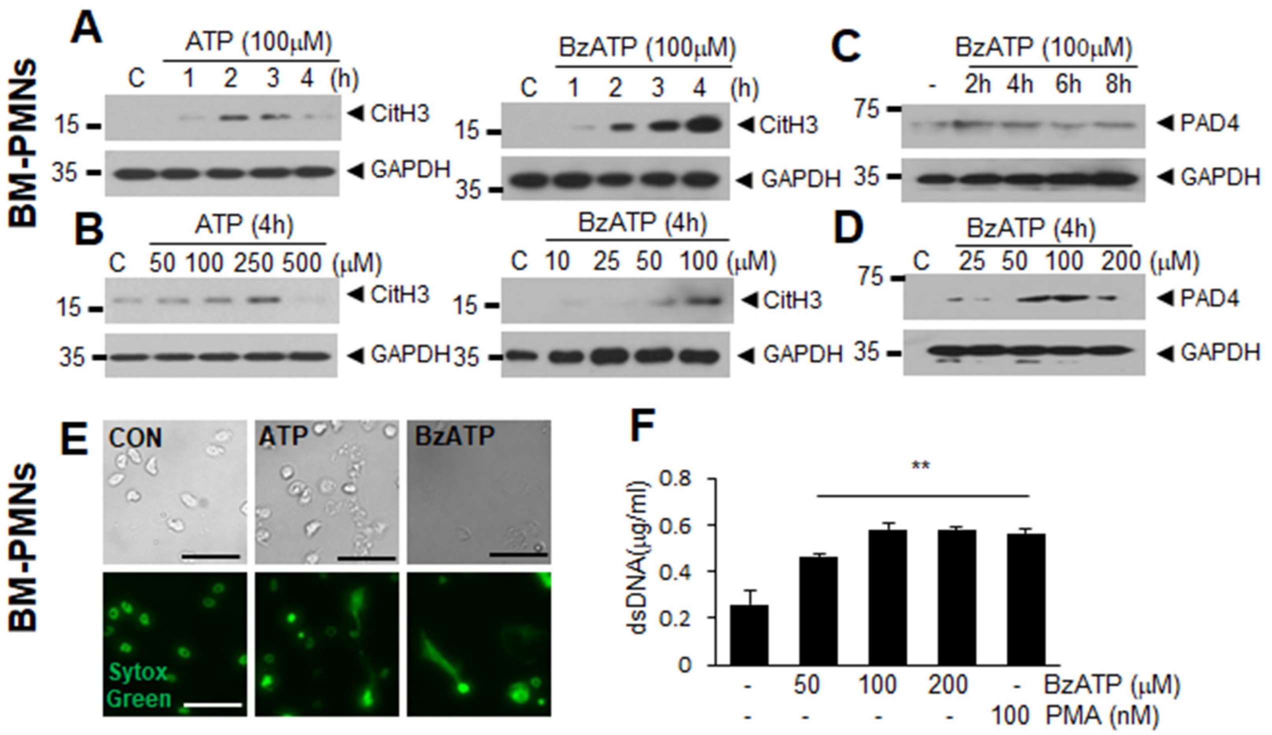

Supplementary Figure 1. ATP induced PAD4 and CitH3 upregulation in bone marrow-derived neutrophils

BM-PMNs were treated with 100 μM of ATP or BzATP for the indicated duration (**a,c**) or with a range of doses of ATP or BzATP for 4 h (**b,d**). Levels of CitH3 (**a,b**) and PAD4 (**c,d**) were determined by immunoblotting (**e,f**). BM-PMNs were treated with 100 μM ATP or BzATP for 4h and dsDNA release was visualized by immunofluorescent with Sytox green (**e**) and the amounts were measured using QuantiT PicoGreen dsDNA reagent (**f**). Scale bars in e represent 50 μm. Results are presented as mean ± SEM (n=3). **p < 0.01 versus the PBS-treated control

Supplementary Figure 2

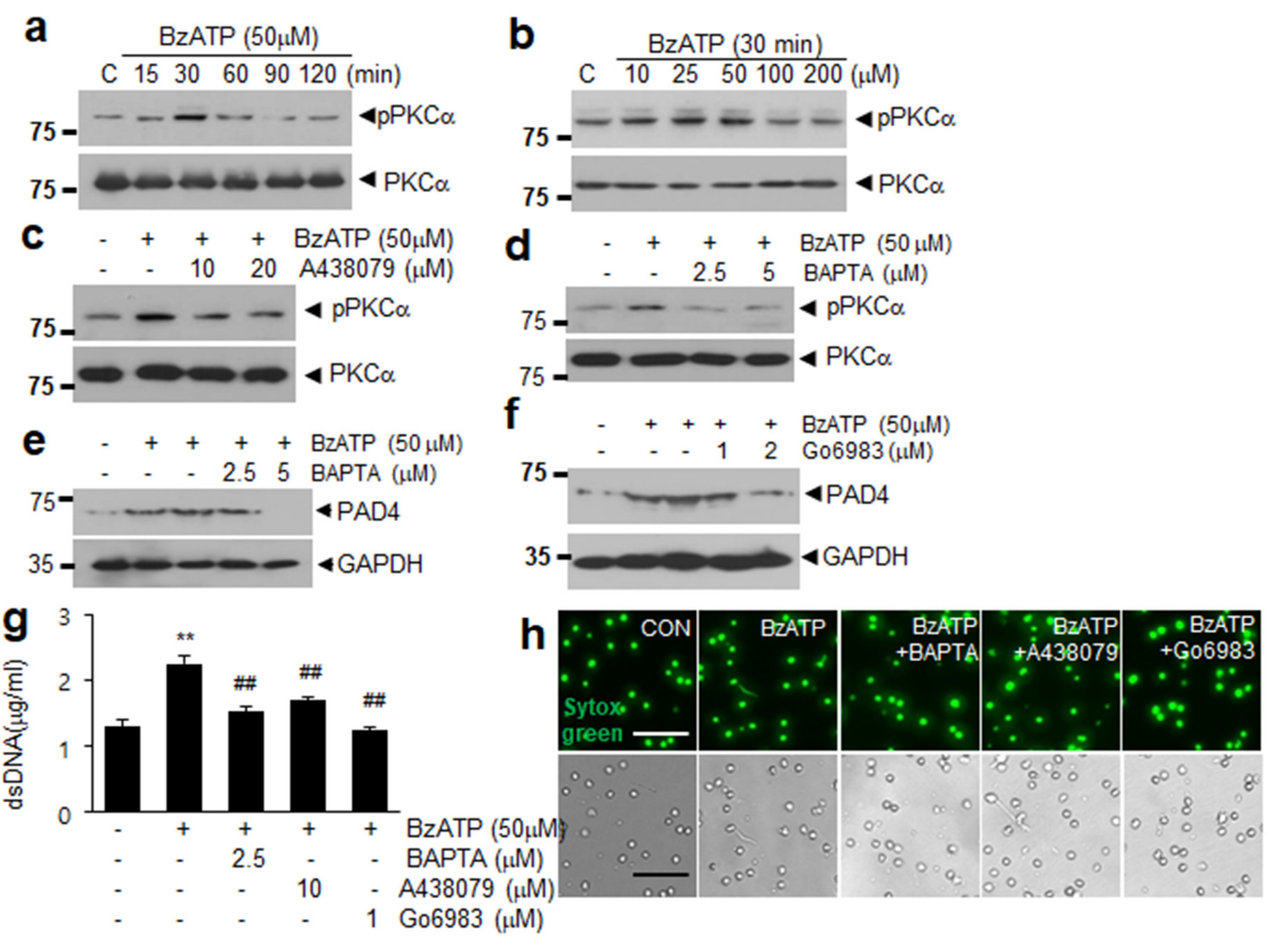

Supplementary Figure 2. PKCα activation in BzATP-P2X7R induced NETosis in blood PMNs

(**a,b**) Blood PMNs were treated with BzATP (50 μM) for the indicated duration or with 10, 20 50, 100, or 200 μM of BzATP for 30 min and levels of PKCα and phospho-PKCα were examined by immunoblotting. (**c,d**) Blood PMNs were pretreated with A438079 (10 or 20 μM) or with BAPTA (2.5 or 5 μM) for 20 min prior to treatment with BzATP (50 μM) for 30 min, then the levels of PKCα and phospho-PKCα were subsequently determined by immunoblotting. (**e,f**) Blood PMNs were pretreated with BAPTA (2.5 or 5 μM) or Go6983 (1 or 2 μM) for 20 min prior to treatment with BzATP (50 μM) for 4 h, then PAD4 levels were subsequently determined by immunoblotting. (**g,h**) Blood PMNs were pretreated with BAPTA (2.5 or 5 μM) or Go8983 (1 μM) for 20 min, and then were treated with 50 μM of BzATP for 18 h, with amounts of dsDNA released subsequently assessed using Quant-iT PicoGreen dsDNA reagent (g) and visualized using Sytox Green (h). Scale bars in h represent 50 μm. Results are presented as mean ± SEM (n=3). **p<0.01 versus the PBS-

treated control, ##$p < 0.01$ versus BzATP-treated cells.

Supplementary Figure 3

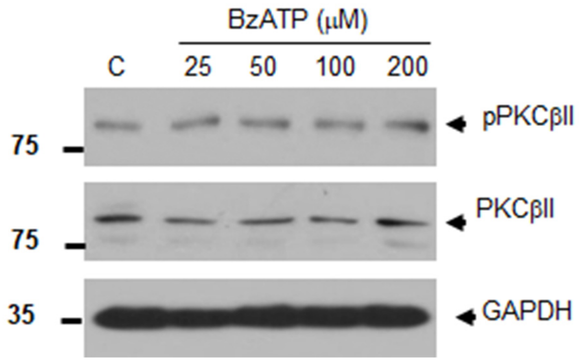

Supplementary Figure 3. PKCβ activation in BzATP-treated blood PMNs

Blood PMNs were treated with BzATP (25, 50, 100, or 200 μM) for 60 min. Levels of PKCβ and phospho-PKCβ were examined by immunoblotting.

Supplementary Figure 4

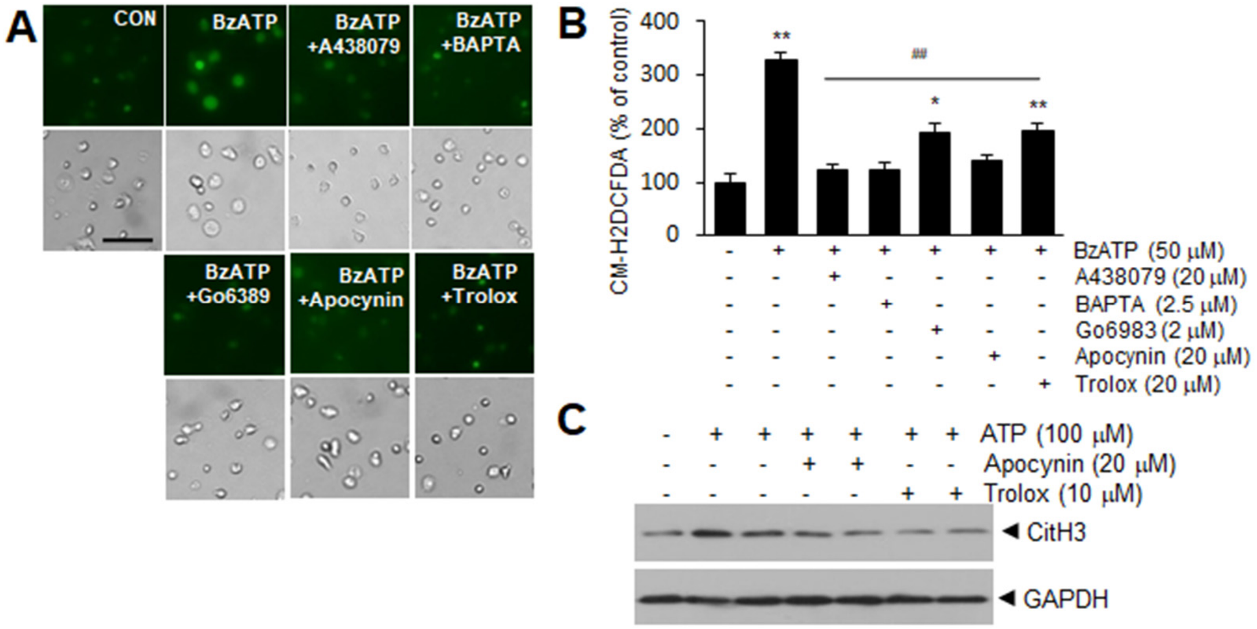

Supplementary Figure 4. Upregulation of ROS production in BzATP-induced NETosis in BM-PMNs

(a,b) BM-PMNs were pretreated with A438079 (20 µM), BAPATA (2.5 µM), Go6983 (2 µM), apocynin (20 µM), or Trolox (20 µM) for 20 min prior to treatment with BzATP (50 µM) for 2 h. Intracellular ROS generation was visualized using CMH2DCFDA and analyzed using ImageJ. (**c**) BM-PMNs were pretreated with 20 µM of apocynin or 10 µM of Trolox for 20 min prior to treatment with ATP (100 µM) for 4 h, with CitH3 levels were subsequently assessed by immunoblotting. Scale bars in a represent 50 µm. Results are presented as mean ± SEM (n=3). $^*p < 0.05$, $^{**}p < 0.01$ versus PBS-treated controls, $^{\#\#}p < 0.01$ versus ATP only-treated cells.
